# Effectiveness and safety of Chinese herbal formula combined with western medicine for ankylosing spondylitis: A protocol for systematic review and meta-analysis

**DOI:** 10.1097/MD.0000000000026374

**Published:** 2021-06-25

**Authors:** Miao Liu, Xiao-Lei Deng, Jing Yu

**Affiliations:** a Dalian Xigang Zhonghe Rheumatism and Immunology Specialist Outpatient Department of Integrated Traditional Chinese Medicine and Western Medicine, Dalian; b Department of Orthopedics and Traumatology; c Department of Rheumatology and Immunology, Affiliated Hospital of Liaoning University of Traditional Chinese Medicine, Shenyang, China.

**Keywords:** ankylosing spondylitis, Chinese herbal formula, meta-analysis, systematic review

## Abstract

**Background:**

Ankylosing spondylitis (AS) is a chronic progressive inflammatory disease of the spine, which mainly invades the sacroiliac joint, spine, and large joints near the trunk, leading to fibrous and skeletal ankylosis and deformity, and can cause damage to the eyes, lung, cardiovascular, kidney and other organs. Chinese herbal formulas (CHF) is an important interventions of Traditional Chinese Medicine (TCM), and CHFs combined with western medicine are widely used in clinical practice to treat AS.

**Methods:**

Eight databases will be systematically retrieved from their inceptions to March 2021. Only randomized controlled trials (RCTs) of CHFs combined with western medicine for AS treatment will meet the inclusion criteria. The primary outcomes we focus on include clinical effectiveness rate, TCM syndrome score, TCM symptom score, Bath ankylosing spondylitis disease activity index (BASDAI), Bath Ankylosing Spondylitis Functional Index (BASFI), Bath Ankylosing Spondylitis Metrology Index (BASMI), chest expansion, nocturnal spinal pain, adverse reactions, erythrocyte sedimentation rate (ESR), and C protein response (CRP). The research screening, data extraction, and risk of bias assessment will be performed independently by 2 researchers, and divergence will be solved by a third researcher. Revman 5.3 software will be used for meta-analysis. The confidence of evidence will be graded using grading of recommendations assessment, development, and evaluation (GRADE) algorithm and methodological quality will be assessed adopting risk of bias in systematic reviews (ROBIS).

**Results:**

This systematic review (SR) will provide evidence-based medical evidence for AS therapy by CHF combined with western medicine and we will submit the findings of this SR for peer-review publication.

**Conclusions:**

This SR will provide latest and updated summary proof for assessing the effectiveness and safety of CHF combined with western medicine for AS.

**Registration number:**

INPLASY 202150089.

## 1 Introduction

Ankylosing spondylitis (AS) is a chronic inflammatory disease of unknown cause that mainly invades the axial bone, characterized by sacroiliac arthritis and adhesions.^[1]^ The disease mostly occurs in adolescents, the age of onset is usually 13 to 31 years, the peak is 20 to 30 years, there is a predilection for males, and the male to female ratio is 3:1.^[2]^ Most patients initially present with sacroiliitis, which progressively involves the lumbar, thoracic, cervical, as well as peripheral joints as the disease progresses and involves multiple systems.^[3]^ AS has an insidious onset with a long disease course and a very high rate of disability. Spinal ankylosis and joint deformities occur in the later course of the disease and the condition is irreversible.^[4]^ Early diagnosis and comprehensive treatment are necessary to improve the prognosis.

AS is an inflammatory disease that primarily involves the axial bones and sacroiliac joints. Other skeletal muscle manifestations of AS are arthritis of the extremities and tendoskeletal enthesitis.^[5]^ Extra articular diseases include anterior uveitis, osteoporosis, heart disease with valvular involvement, kidney disease, lung disease, gastrointestinal disease, and skin disease.^[6]^ The initial stage of AS was dominated by sacroiliitis, after which the inflammation gradually involved the lumbar, thoracic, and cervical spine, and finally formed the result that the lumbar and dorsal plates were straight and stiff or bent and could not be upright, accompanied by pain manifestations throughout the course of the disease.^[6]^ In addition, it is often accompanied by lesions elsewhere, which can lead to acute iritis, thoracic sclerosis, pulmonary fibrosis, aortic regurgitation, hematuria, and proteinuria.^[7]^

The etiology of AS remains undefined, but studies have found that it is highly correlated with human leukocyte antigen (HLA)-B27.^[8]^ HLA-B27, the major histocompatibility complex class I allele, is widely distributed in human populations depending on ethnicity, and the incidence of AS is correlated with the frequency of HLA-B27 in different populations.^[9]^ Therefore, AS can be basically defined as a genetic disease. The prevalence of HLA-B27 alleles approaches 90%; however, the pathogenic mechanism underlying this potential association remains unclear, and the mechanisms that have been proposed include the arthritis gene peptide theory, HLA-B27 heavy chain dimer formation, and HLA-B27 misfolded and unfolded protein response.^[10]^ In addition, studies have shown that people with metabolic diseases such as obesity and diabetes are at high risk of AS.^[11]^ Studies have shown that infection is also involved in the pathogenesis of AS.^[12]^ Gastrointestinal and respiratory infections often occur in the development of the disease.^[13]^ The infected bacteria include klebsiella pneumoniae, salmonella, shigella, yersinia, campylobacter, chlamydia and mycoplasma.^[14]^ Most of the patients with AS have elevated immunoglobulin. Immunosuppressive agents have a good effect on the treatment of the disease.^[14]^ Therefore, immune factors are an important cause of AS. Endocrine factors are also involved in the pathogenesis of AS. The high incidence of male indicates that androgen may be one of the pathogenic factors of as. Besides, the abnormality of some cytokines, leptin, type II collagen, and proteoglycan are also related to the pathogenesis of AS.^[14]^

The disease activity of AS has a great impact on the decline of quality of life and the loss of working ability, but there is no drug can cure the disease. The treatment goal of patients with AS is to relieve the symptoms of spinal or systemic pain and morning stiffness, restore and maintain the patient's activity function to the greatest extent, prevent spinal and joint deformity, improve the social activity ability and work ability, and improve the quality of life.^[15]^

AS is mainly treated with a combination of nonpharmacological and pharmacological modalities. Nonpharmacological treatments mainly use physical therapy, exercise therapy and health management, etc, to maintain and promote joint mobility function.^[16]^ Exercise is the basis of nonpharmacologic interventions, and exercise may improve pain, physical function, spinal mobility, and the patient's overall assessment.^[17]^ The western medicine treatment mainly includes nonsteroidal anti-inflammatory drugs (NSAIDs), immunosuppressive agents, glucocorticoids, and so on. NSAIDs are the first-line treatment for AS.^[18]^ Traditional antirheumatic drugs such as methotrexate and sulfasalazine are not effective or efficacious for axial spondyloarthritis.^[19]^ TNF-a inhibitors are the second line of choice for the treatment of AS, and multiple TNF-a inhibitors have proven efficacy for active AS.^[20]^ However, some patients have to discontinue the treatment because of the obvious side effects of western medicine.^[21]^ The study of AS in Traditional Chinese Medicine (TCM) has a long history and has accumulated extensive experience in treating AS, and some Chinese herbal formulas (CHF) have been confirmed to have better clinical effects on this disease.^[22]^ On the basis of the holistic concept and syndrome differentiation, TCM can be directed at different pathogenic links, using a variety of methods, and through multiple pathways, to achieve the effect of treating diseases and regulating immunity, and can alleviate the side effects of western medicine.^[23]^ Therefore, integrated TCM and western medicine in the treatment of AS can synergize with each other to exert their own advantages, which can both remove multiple pathogenic factors of AS and improve the systemic status to achieve the best therapeutic effect.^[24]^ At present, a host of clinical trials have shown that CHF combined with western medicine has good clinical effectiveness and safety in the treatment of AS.^[25]^

Systematic review is the process of applying a clear methodology to find, select, and critically appraise relevant studies, from which data are extracted and combined using appropriate statistical methods to reach a comprehensive conclusion, with a view to providing evidence to address a specific clinical question.^[26]^ Meta-analysis is the process of comprehensively collecting all relevant studies and critically evaluating and analyzing them one by one, and statistically processing the data with the method of quantitative synthesis to reach a comprehensive conclusion.^[27]^ Meta-analysis can overcome the inadequacy of traditional literature reviews and provide a quantitative synthesis, which can provide a systematic, reproducible, and objective synthesis method for the question. In addition, meta-analysis has the advantages of increasing test power, increasing study precision, answering questions not posed by a single study, resolving controversies arising from conflicting findings, or generating new hypotheses.^[28]^ Therefore, we will implement a systematic review and meta-analysis to synthesize clinical trials of CHFs combined with western medicine for AS treatment, and conduct a quantitative synthesis to evaluate its efficacy and safety.

## 2 Methods and analysis

### 2.1 Objective

This systematic review and meta-analysis aims to comprehensively synthesize randomized controlled trial (RCT) of CHFs combined with western medicine for the treatment of AS to assess their efficacy and safety.

### 2.2 Study registration

This systematic review has been registered on International Platform of Registered Systematic Review and Meta-analysis Protocols (INPLASY no. 202150089, https://inplasy.com/?s=202150089). This study will be reported in accordance with Preferred Reporting Item for Systematic Review and Meta-analysis Protocols (PRISMA-P) guidance to guarantee the quality of reporting of this protocol.^[29]^

### 2.3 Inclusion and exclusion criteria

#### 2.3.1 Type of study

We will include RCTs of CHFs combined with western medicine in the treatment of AS, which should evaluate at least one primary outcome. Only Chinese and English literatures will be included in this study regardless of blind and publication type. Observational study, Quasi-RCTs, duplicated publications, animal researches, narrative publications, case reports, editorials, and pharmacological experiments will be excluded.

#### 2.3.2 Type of participants

The participants should be diagnosed with AS by using clearly defined or internationally recognized criteria. No restrictions will be placed on race, gender, educational background, and duration of AS.

#### 2.3.3 Type of interventions

CHFs combined with western medicine should be applied in the treatment group. No restrictions will be applied to the composition, dosage, and course of Chinese medicine. The same western medicine should be used for the control group. CHFs combined with other TCM interventions such as acupuncture and Qigong will be excluded. Western medicines are those recommended by the guidelines or expert consensus, including NSAIDs, TNF receptor blockers, antirheumatic drugs, corticosteroids, etc, for example, ibuprofen, adalimumab, sulfasalazine, Methotrexat, leflunomide, etc.

#### 2.3.4 Type of outcomes

The outcome measures we focused on included clinical effectiveness rate, TCM syndrome score, TCM symptom score, Bath ankylosing spondylitis disease activity index (BASDAI), functional ability that measured by the Bath Ankylosing Spondylitis Functional Index (BASFI), mobility that measured by the Bath Ankylosing Spondylitis Metrology Index (BASMI), Chest expansion, nocturnal spinal pain, adverse reactions, erythrocyte sedimentation rate (ESR), and C protein response (CRP).

### 2.4 Search strategy

RCTs of CHFs combined with western medicines for AS will be retrieved in the following databases: PubMed, EMBASE, Cochrane Central Register of Controlled Trials, Web of Science, Chinese Biomedical Literatures Database (CBM), China National Knowledge Infrastructure (CNKI), Wang Fang Database (WF), Chinese Scientific Journal Database (VIP). The retrieval time range is from their inceptions of each database to March 2021. Subject words combined with free words method will be used for retrieval. The language is limited to Chinese and English. The search strategy of PubMed can be found in supplemental digital content (Supplemental Digital Content); additionally, similar search strategy details will also be applied to any other electronic databases. WHO clinical trials registry, Clinical Trials.gov trials registry, and Chinese Clinical Trial Registry will also be retrieved to confirm ongoing or completed clinical trials. Furthermore, reference lists of all eligible trials and relevant reviews will be searched.

### 2.5 Studies Selection

**Figure 1:**
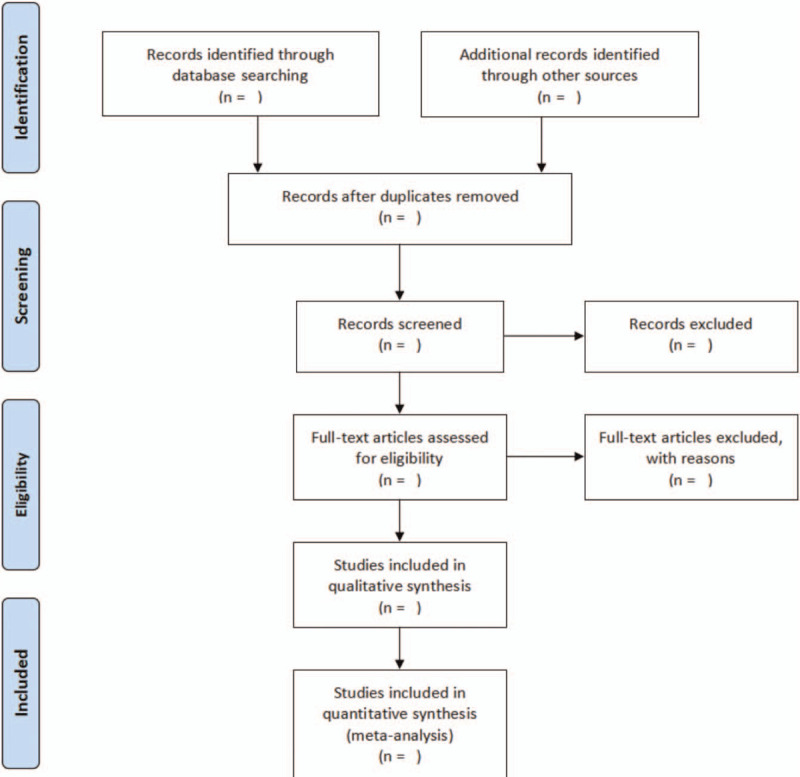
Flow diagram of study selection process. Details of the selection process will be presented in the PRISMA flow chart.

According to the search strategy of this article, the search and screening of the literature will be performed independently by 2 reviewers. First, the researcher will import the retrieved literatures into the Note Express 3.2 software and remove duplicates, and then read the titles and abstracts of the literatures one by one to download the full text of the literatures that met the inclusion criteria. Second, literature screening will be performed according to the developed inclusion and exclusion criteria and the final included literatures will be identified, and disagreements will be resolved by mutual discussion or assisted by a third investigator. Details of the selection process will be presented in the PRISMA flow chart (Fig. 1).

### 2.6 Data extraction

After identifying the included articles, 2 investigators will independently extract information based on a pre-designed form, which included 4 aspects: general information (author, publication source, sample size, age, gender, baseline data, diagnostic criteria, inclusion and exclusion criteria), methods (randomization and allocation concealment; blinding; withdrawals and lost to follow-up), interventions (interventions in treatment and control groups, duration, follow-up time), and outcomes (type of indicators and methods of determination). If articles included did not specifically describe or relevant information was missing, authors will be contacted for further information. Any disagreements arising from this process will be resolved by consensus or adjudicated by a third senior investigator.

### 2.7 Risk of bias assessment

The risk of bias of RCTs included will be evaluated on the basis of the risk of bias tool described in the Cochrane Handbook for Systematic Reviews of Interventions.^[30]^ Two investigators will independently evaluate the risk of bias from 6 aspects: random sequence generation, allocation consensus, blinding methods, complete outcome data, selective outcome reporting, and other bias. A research with a high risk of bias in one or more domains will be viewed as high risk of bias. A study with a low risk of bias in all domains will be considered as low risk of bias. If not, a research will be treated as unclear risk of bias.

Furthermore, 2 reviewers will independently use the modified Jadad scale to assess the quality of the RCTs included.^[31]^ Four domains will be appraised, namely “randomization,” “concealment of allocation,” “double blinding,” “withdrawals and dropouts”. According to the scoring principle, 1 to 3 scores will be viewed as low-quality literature, and 4 to 7 scores as high-quality literature.

Disagreements encountered during this process will be resolved through discussion or by a third senior investigator.

### 2.8 Strategy for data synthesis

RevMan V.5.3.0 software (The Nordic Cochrane Center, The Cochrane Collaboration, 2014, Copenhagen, Denmark) will be used for meta-analysis. Binary variables will be expressed using the risk ratio (RR) with 95% confidence interval (CI) and continuous variables by the weighted mean difference (WMD) with 95% CI. The Q-test and *I*^*2*^ values will be applied to measure the inter-study heterogeneity. When the *P*-value of Q-test > .1 and *I*^*2*^ < 50%, heterogeneity is acceptable and a fixed effects model will be applied. If *P*-value of Q-test < .1 and *I*^*2*^ > 50%, heterogeneity is significant and subgroup analysis will be developed to investigate the possible sources according to the characteristics of study, types of intervention and controls, different outcome measurements and study quality, if still unable to find, we will use random effect model to estimate or descriptive analysis.

### 2.9 Sensitivity analysis

The sensitivity analysis is to examine the stability of the results of the corresponding analysis to illustrate the reliability of the conclusions of the study by considering different possible conditions. Sensitivity analysis will be performed by varying certain important factors that may affect the results such as inclusion criteria, study quality, statistical methods and effect size, etc, and then we will perform the analysis to compare with the original results. In case of consistency, the original result is suggested to be stable and the conclusion is reliable, and conversely, the result is unstable and the conclusion is unreliable.

### 2.10 Publication biases

If the included literatures >10, we will employ funnel plot analysis for potential publication bias. The existence of publication bias will be judged by analyzing the distribution patterns of the collected clinical study data. Funnel plots are drawn with the effect size RR as the abscissa and the study sample size as the ordinate, and studies with small sample sizes, low precision, distributed at the bottom of the funnel plot, and scattered toward the periphery. Studies with large sample sizes, high precision, are distributed on the top of the funnel plot, and are centered toward the middle. When publication bias is not present, its shape resembled a symmetrically inverted funnel.

### 2.11 Rating the confidence in estimates of the effect

Systematic reviews and meta-analyses are secondary studies conducted on completed studies, which produce new findings whose quality is greatly influenced by the quality of original studies, and new evidence whose credibility requires further evaluation. We will grade the new evidence generated by this systematic review using the GRADE profile 3.6.1 tool and report its results in detail.^[32]^

### 2.12 Assessment of methodological quality

We will adopt the Risk of Bias in Systematic Reviews tool to assess the methodological quality of this systematic review, which has shown good reliability and validity.^[33]^ This tool mainly consists of 3 major evaluation stages, the first one is to evaluate the relevance of the research to the topic. The second stage is to assess the possible introduced risk of bias in the course of the study (inclusion criteria, screening of the literature, data collection and evaluation, and data synthesis and main conclusions). The third stage is to judge the impact of the risk of bias present in the study on the overall conclusions. During the evaluation process, the responses to the iconic questions will be expressed as “Yes” “Probably Yes,” “Probably No” “No,” “No information,” and if one is rated as “Probably No” or “No,” the overall evaluation of this domain will viewed as high risk.

### 2.13 Reporting criteria

This systematic review and meta-analysis will be reported in accordance with Preferred Reporting Items for Systematic Reviews and Meta-Analyses Extension for Chinese Herbal Medicines 2020 (PRISMA-CHM 2020).^[34]^

### 2.14 Ethics and dissemination

This systematic review will not require ethical approval because there are no data used in our study that are linked to individual patient data. In addition, findings will be disseminated through peer-review publications.

## 3 Discussion

AS is a chronic progressive inflammatory change of the spine, which mainly invades the sacroiliac joint, the spine, and the large joints near the trunk, leading to fibrous and skeletal ankylosis and deformity, and can damage the eyes, lungs, cardiovascular, kidney, and other organs in varying degrees.^[35]^ AS is a systemic disease, which can have anorexia, low fever, fatigue, weight loss, and mild anemia. The majority of AS has its onset in young adulthood, the onset is rare beyond the age of 40 years, and the development of lesions is slow in female patients, often with delayed diagnosis.^[36]^ Lower back pain and spinal rigidity are the most common manifestations of AS. Lower back pain occurs slowly, is dull aching, and sometimes involves the buttocks. Morning stiffness is an extremely common symptom and can last for up to hours. There is chest pain with radiating intercostal neuralgia as the lesion progresses to the thoracic spine involving the costospinal joints. AS that continues to progress will causes thoracic kyphosis and cervical spine morbidity. In terms of treatment, TCM and Western medicine have their own good points. CHF is an important intervention measure of TCM. CHFs combined with western medicine in the treatment of AS can treat the whole body and local lesions of patients. The 2 intervention measures can play a synergistic role, enhance efficiency, and reduce toxicity, so as to obtain satisfactory results.

## Author contributions

M L, X-L D, and Y J participated in the conception and design of the study, including search strategy development. M L, X-L D tested the feasibility of the study and wrote the manuscript. All the authors critically reviewed this manuscript and approved the final version.

**Conceptualization:** Miao Liu, Xiao-Lei Deng, Jing Yu.

**Formal analysis:** Miao Liu, Xiao-Lei Deng.

**Funding acquisition:** Jing Yu.

**Investigation:** Miao Liu, Xiao-Lei Deng.

**Methodology:** Miao Liu, Xiao-Lei Deng, Jing Yu.

**Project administration:** Miao Liu, Xiao-Lei Deng.

**Writing – original draft:** Miao Liu, Xiao-Lei Deng.

**Writing – review & editing:** Miao Liu, Xiao-Lei Deng, Jing Yu.

## Supplementary Material

Supplemental Digital Content
