# Supplementary material for: Effectiveness and safety of Chinese herbal formula combined with western medicine for ankylosing spondylitis: A protocol for systematic review and meta-analysis
Source: Medicine (Baltimore). 2021 Jun 25;100(25):e26374. doi: 10.1097/MD.0000000000026374 (PMC8238346; doi:10.1097/MD.0000000000026374)
Supplement: Supplemental Digital Content [file medi-100-e26374-s001.docx]

| **Search Strategy in Pubmed:** (揗edicine, Chinese Traditional?[MeSH Terms] OR 揷hinese medic*?[Title/Abstract] OR 揾erb*?[Title/Abstract] OR 揹ecoction?[Title/Abstract] OR 揷hinese patent medicine?[Title/Abstract] OR 搕raditional chinese medicine prescription?[Title/ Abstract] OR 搕raditional chinese compound?[Title/Abstract]) AND (“Spondylitis, Ankylosing”[MeSH Terms] OR 揂nkylosing Spondylitis?[Title/Abstract] 揝pondyloarthritis?[Title/Abstract] OR 揝pondyloarthropathies?[Title/Abstract] OR 揝eronegative Spondyloarthropathies?[Title/Abstract]) AND (“randomized controlled trial”[Publication Type]) OR “controlled clinical trial” [Publication Type]) OR “randomized” [Title/Abstract]) OR “randomly” [Title/Abstract]) OR “drug therapy” [MeSH Subheading]) OR “trial” [Title/ Abstract]) |
| --- |
